# Virulence gene detection and antimicrobial resistance analysis of* Enterococcus faecium* in captive giant pandas (*Ailuropoda melanoleuca*) in China

**DOI:** 10.1186/s13028-023-00668-z

**Published:** 2023-02-03

**Authors:** Hai-Feng Liu, Xiao-Yao Huang, Zhe-Meng Li, Zi-Yao Zhou, Zhi-Jun Zhong, Guang-Neng Peng

**Affiliations:** Department of Veterinary Surgery, College of Veterinary Medicine, Sichuan Agricultural University, Chengdu, 611130 People’s Republic of China

**Keywords:** Drug resistance, *Enterococcus faecium*, Giant panda, Virulence gene

## Abstract

**Background:**

The emergence of multidrug resistance among enterococci makes effective treatment of *enterococcal* infections more challenging. Giant pandas (*Ailuropoda melanoleuca*) are vulnerable to oral trauma and lesions as they feast on bamboo. *Enterococci* may contaminate such oral lesions and cause infection necessitating treatment with antibiotics. However, few studies have focused on the virulence and drug resistance of oral-derived enterococci, including *Enterococcus faecium*, in giant pandas. In this study, we analyzed the prevalence of 8 virulence genes and 14 drug resistance genes in *E. faecium* isolates isolated from saliva samples of giant pandas held in captivity in China and examined the antimicrobial drug susceptibility patterns of the *E. faecium* isolates.

**Results:**

Twenty-eight isolates of *E. faecium* were successfully isolated from the saliva samples. Four virulence genes were detected, with the *acm* gene showing the highest prevalence (89%). The *cylA*, *cpd*, *esp*, and *hyl* genes were not detected. The isolated *E. faecium* isolates possessed strong resistance to a variety of drugs; however, they were sensitive to high concentrations of aminoglycosides. The resistance rates to vancomycin, linezolid, and nitrofurantoin were higher than those previously revealed by similar studies in China and other countries.

**Conclusions:**

The findings of the present study indicate the drugs of choice for treatment of oral *E. faecium* infection in the giant panda.

## Background

Giant pandas (*Ailuropoda melanoleuca*) are vulnerable to wear of their teeth, flapping, and deformation as they eat bamboo. This diet leads to an increased risk of oral trauma and lesions, including caries and dental disease [1]. The occurrence and spread of oral diseases among giant pandas, as well as their general health, are closely related to the oral flora [2]. *Enterococcus faecium* may cause severe disease that can be challenging to treat because of antibiotic resistance [3, 4]. However, few reports to date have focused on the oral flora of giant pandas, especially with respect to virulence and antibiotic resistance.

*Enterococcus faecium* is a Gram-positive bacterium that is widely distributed in nature. It is also an opportunistic pathogen and one of the most common causes of nosocomial infections in humans [5]. The virulence of *E. faecium* is related to production of substances such as cytolysin, aggregation substance, surface protein, and gelatinase as well as to biofilm formation [6]. Because of the broad virulence of *E. faecium* and its ability to spread resistance genes, gaining further knowledge of the virulence genes and drug resistance patterns of this bacterium is important for both human and animal health. *Enterococcus faecium* may migrate though the oral mucosa and cause bacteriemia; systemic disease; urinary tract infections; abdominal, pelvic, and soft tissue infections; and endocarditis [7]. Multilocus sequence typing, amplified fragment length polymorphisms, and pulsed-field gel electrophoresis have been employed to investigate the molecular epidemiology of *E. faecium* isolates from various sources. The large phenotypic heterogeneity among different isolates of *E. faecium* may help to detect antibiotic-resistant isolates and track their spread. There are two major genomic groups of *E. faecium* (groups I and II) that have different origins, safety profiles, and antibiotic resistance and virulence profiles and therefore distinct abilities to cause clinical outbreaks [8, 9].

In this study, we analyzed the prevalence of 8 virulence genes and 14 drug resistance genes in *E. faecium* isolates isolated from saliva samples of giant pandas held in captivity in China and examined the antimicrobial drug susceptibility patterns of the *E. faecium* isolates.

## Methods

### Bacterial isolates

The isolates were isolated from the sublingual saliva of 30 healthy giant pandas held at the Giant Panda Breeding Research Base in Chengdu, Sichuan Province, China. The sample population consisted of 15 juvenile giant pandas (8–9 months old) and 15 adult giant pandas (6–10 years old). The saliva specimens were spread on solid Luria–Bertani (LB) medium and *Enterococcus* CHROMagar™ chromogenic medium plates, which were then incubated at 37 °C for 18 to 24 h. A total bacterial DNA extraction kit (Tiangen Biochemical Technology, Beijing, China) was used to extract the DNA template from purified colonies. Extracted DNA was stored at − 20 °C, and the 16S rRNA gene was amplified from all isolates according to a previous report [10].

### Sequence and data analysis

The 16S universal primers were used to amplify a sequence of approximately 1500 base pairs of the target gene, and the polymerase chain reaction (PCR) products of the isolated isolates were sent to Bioengineering Co., Ltd. (Shanghai, China) for 16S rRNA sequencing. The sequencing data were compared with sequences in the GenBank database using the BLAST search tool available on the NCBI website (http//www.ncbi.nlm.nih.gov/). Sequence homology higher than 98% was considered to indicate isolate similarity according to a previous report [11].

### Virulence gene analysis

The *E. faecium* virulence genes *cylA*, *cpd*, *asa1*, *ace*, *acm*, *esp*, *gelE*, and *hyl* were detected by multiplex PCR and single-round PCR according to previous reports [12, 13]. The primer sequences and product lengths are shown in Table 1.

**Table 1:** *Enterococcus faecium* virulence gene primer sequence and product length

Target genes	Primer sequence (5′–3′)	Product size (bp)	Ta ( °C)
*cylA*	ACTCGGGGATTGATAGGC GCTGCTAAAGCTGCGCTT	688	56
*cpd*	TGGTGGGTTATTTTTCAATTC TACGGCTCTGGCTTACTA	782	50
*asa1*	GCACGCTATTACGAACTATGA TAAGAAAGAACATCACCACGA	375	51
*ace*	GGAATGACCGAGAACGATGGC GCTTGATGTTGGCCTGCTTCCG	616	51
*acm*	GGCCAGAAACGTAACCGATA CGCTGGGGAAATCTTGTAAA	353	51
*esp*	AGATTTCATCTTTGATTCTTGG AATTGATTCTTTAGCATCTGG	510	56
*gelE*	AATTGCTTTACACGGAACGG GAGCCATGGTTTCTGGTTGT	548	51
*hyl*	ACAGAAGAGCTGCAGGAAATG GACTGACGTCCAAGTTTCCAA	276	56

The *cylA*, *esp*, and *hyl* genes were amplified by multiplex PCR using the following reaction mix: 25 μL of Taq^™^ version 2.0 plus dye, 5 μL of DNA template, 0.1 μM of each of the upstream and downstream specific primers for *hyl*, 0.2 μM of each of the upstream and downstream specific primers for *cylA* and *esp*, and double-distilled water (ddH_2_O) up to 50 μL. The multiplex PCR cycle conditions involved a denaturation step at 95 °C for 15 min followed by 30 cycles of 94 °C for 1 min, 56 °C for 1 min, and 72 °C for 1 min, and a final extension step at 72 °C for 10 min.

The single-round PCR reaction mix comprised 12.5 μL of Taq^™^ version 2.0 plus dye, 2 μL of DNA template, 1 μL of each of the upstream and downstream specific primers, and ddH_2_O up to 25 μL. The single-round PCR cycle conditions involved a denaturation step at 94 °C for 5 min followed by 30 cycles of 94 °C for 30 s, annealing at an appropriate temperature for 30 s and 72 °C for 10 s, and a final extension step at 72 °C for 1 min.

### Resistance gene analysis

Isolates were analyzed by PCR for the β-lactam resistance genes (*blaTEM*, *blaSHV*, and *blaCTX-M*), oxazolidinone resistance genes (*oprtA*, *cfr*, and *poxtA*), aminoglycoside resistance genes (*aac(6′)-aph(2′′)*, *aph(2′′)-Ib*, *aph(2′′)-Ic*, and *aph(2′′)-Id*), glycopeptide resistance genes (*vanA* and *vanB*), and macrolide resistance genes (*ermA* and *ermB*)*.* Primer sequences and product lengths are shown in Table 2.

**Table 2:** *Enterococcus faecium* resistance gene primer sequence and product length

Target genes	Primer sequence (5′–3′)	Product size (bp)
*blaTEM*	CCCCGAAGAACGTTTTC ATCAGCAATAAACCAGC	516
*blaSHV*	TCAGCGAAAAACACCTTG TCCCGCAGATAAATCACCA	475
*blaCTX-M*	TCAGCGAAAAACACCTTG GATATCGTTGGTGGTGCCAT	543
*optrA*	AGGTGGTCAGCGAACTAA ATCAACTGTTCCCATTCA	1395
*cfr*	TGAAGTATAAAGCAGGTTGGGAGTCA ACCATATAATTGACCACAAGCAGC	746
*poxtA*	GGTGGATTTACCGACACCGT GACCAGTGGAAATGCCCGTA	943
*aac(6*′*)-aph(2*′′*)*	GAGCAATAAGGGCATACCAAAAATC CCGTGCATTTGTCTTAAAAAACTGG	505
*aph(2*′′*)-Ib*	TATGGATTCATGGTTAACTTGGACGCT GAG ATTAAGCTTCCTGCTAAAATATAAACA TCTCTGCT	906
*aph(2*′′*)-Ic*	GAAGTGATGGAAATCCCTTCGTG GCTCTAACCCTTCAGAAATCCAGTC	627
*aph(2*′′*)-Id*	GGTGGTTTTTACAGGAATGCCATC CCCTCTTCATACCAATCCATATAACC	642
*vanA*	GGGAAAACGACAATTGC GTACAATGCGGCCGTTA	732
*vanB*	ATGGGAAGCCGATAGTC GATTTCGTTCCTCGACC	635
*ermA*	TCTAAAAAGCATGTAAAAGAA CGATACTTTTTGTAGTCCTTC	645
*ermB*	GAAAAGGTACTCAACCAAATA AGTAACGGTACTTAAATTGTTTA	639

The detection of drug resistance genes was performed using single-round PCR. The PCR reaction mix comprised Taq^™^ version 2.0 plus 12.5 μL of dye, 2 μL of DNA template (Table 3), 1 μL each of the upstream and downstream specific primers, and ddH_2_O up to 25 μL.

**Table 3:** Drug-resistant gene of polymerase chain reaction (PCR) procedure

Target genes	Ta ( °C)	PCR procedure
*bla*TEM	49	95 °C 2 min + {95 °C 1 min + 49 °C 1 min + 72 °C 1 min} [47] × 35 + 72 °C 7 min
*bla*SHV	53	95 °C 3 min + {95 °C 1 min + 53 °C 1 min + 72 °C 1 min} × 35 + 72 °C 7 min
*bla*CTX-M	51	94 °C 2 min + {94 °C 30 s + 51 °C 30 s + 72 °C 30 s} × 35 + 72 °C 3 min
*optrA*	50	
*cfr*	56	95 °C 5 min + {94 °C 30 s + 1 min + 72 °C 1 min} × 30 + 72 °C 5 min
*poxtA*	58	
*aac(6')-aph(2'')*	61	
*aph(2'')-Ib*	55	94 °C 10 min + {94 °C 1 min + 1 min + 72 °C 1 min} × 30 + 72 °C 10 min
*aph(2'')-Ic*	55	
*aph(2'')-Id*	53.4	
*van*A	54	94 °C 2 min + {94 °C 1 min + 54 °C 1 min + 72 °C 1 min} × 30 + 72 °C 10 min
*van*B	54	
*erm*A	52	93 ℃ 3 min + {93 °C 1 min + 52 °C 1 min + 72 °C 1 min} × 35 + 72 °C 5 min
*erm*B	52	

β-lactam resistance genes: *bla*TEM, *bla*SHV and *bla*CTX-M; Linezolid resistance gene: *cfr*, *optrA* and *poxtA*; Aminoglycoside resistance genes: *aac(6')-aph(2")*, *aph(2")-Ib*, *aph(2")-Ic* and *aph(2")-Id*; Vancomycin resistance gene: *van*A and *van*B; Erythromycin resistance gene: *erm*A and *erm*B

### Antimicrobial susceptibility test

The susceptibility of all isolates to 10 antibiotics was analyzed by the standard disk diffusion method [14]. The results were determined according to the diameter of the bacteriostatic ring and the CLSI (2018) Standard Guidelines. Isolates were classified as sensitive (S), intermediate (I), or resistant (R) (Table 4).

**Table 4:** Antibiotic susceptibility test standard

Class of antibiotics	Antibiotic	Inhibition zone diam		
		S	I	R
Beta-lactamase	P	≥ 15	–	≤ 14
	AM	≥ 17	–	≤ 16
Oxazolidinones	LZD	≥ 23	21–22	≤ 20
Aminoglycoside	S300	≥ 10	7–9	≤ 6
	GM120	≥ 10	7–9	≤ 6
Glycopeptides	VAN	≥ 17	15–16	≤ 14
Macrolides	E	≥ 23	14–22	≤ 13
Quinolones	LEV	≥ 17	14–16	≤ 13
	CIP	≥ 21	16–20	≤ 15
Nitrofurans	FD	≥ 17	15–16	≤ 1

P Penicillin, *AM* Ampicillin, *LZD* Linezolid, S300 High level streptomycin, *GM120* High level gentamicin, *VAN* Vancomycin, E Erythromycin, *LEV* Levofloxacin, *CIP* Ciprofloxacin, *FD* Nitrofurantoin

## Results

### Identification of E. faecium

On LB nutrient agar medium, the *E. faecium* colonies appeared gray-white and translucent with rounded protrusions, smooth surfaces, and well-demarcated. On CHROMagar™ chromogenic medium, the *Enterococcus* colonies appeared purple. Microscopy of Gram-stained slides revealed the presence of Gram-positive cocci.

### PCR amplification and electrophoretic identification of the 16S rRNA

In total, 28 isolates of *E. faecium* were successfully identified after comparison with sequences in the GenBank database.

### Identification of virulence genes

**Fig. 1:**
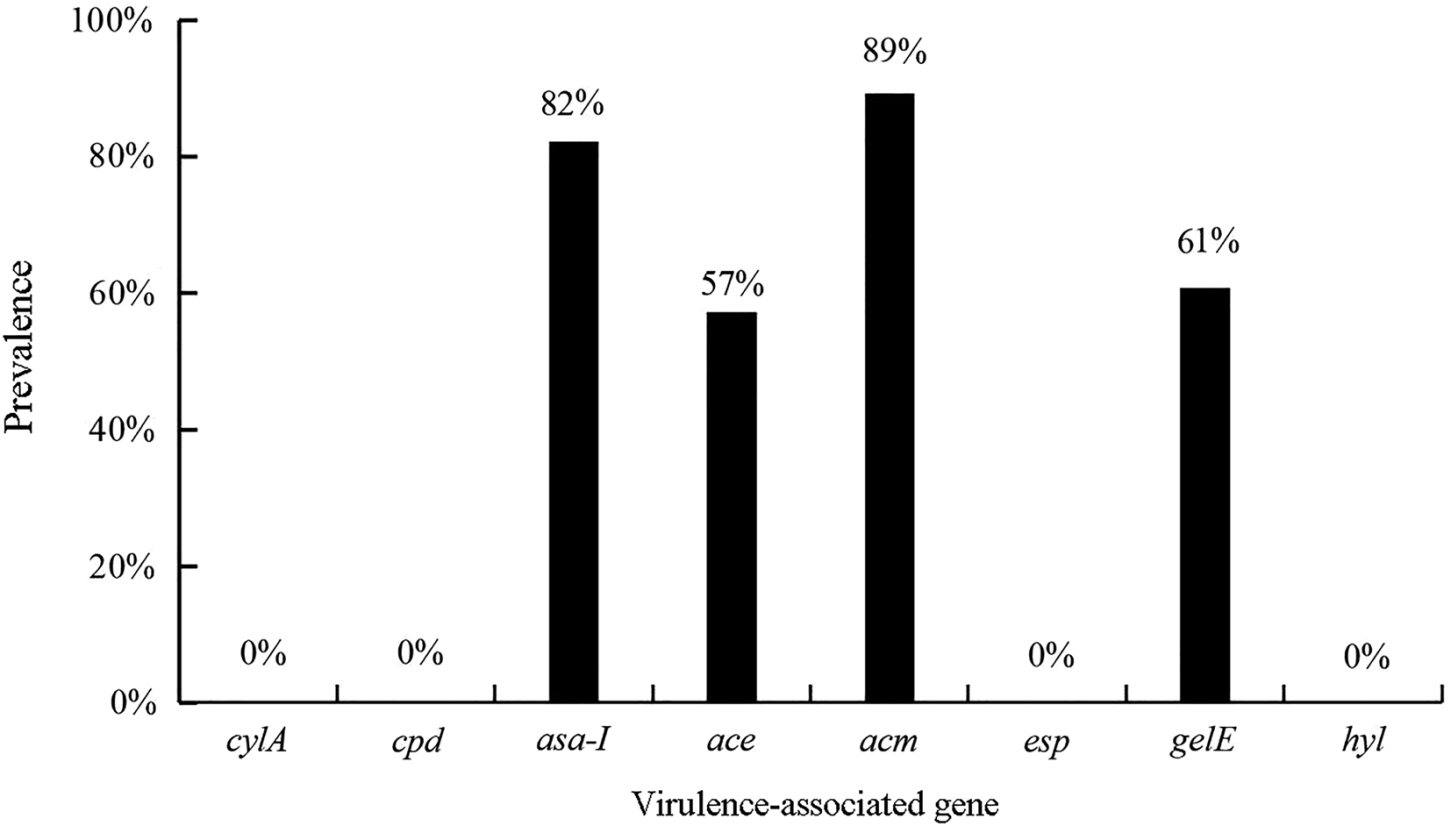
Prevalence of virulence-associated genes in 28 isolates of *Enterococcus faecium cylA* cytolysin, cpd: sex pheromones; *asa1* aggregation substance, *ace/acm* adhesin to collagen, *esp* enterococcal surface protein, *gelE* gelatinase, *hyl* hyaluronidase

Eight virulence genes were detected, of which four were the *ASA1*, *ACE*, *ACM*, and *gelE* genes. The *cylA*, *cpd*, *esp*, and *hyl* genes were not detected in the 28 isolates of *E. faecium*. Among the 28 isolates of *E. faecium*, the adhesin gene *acm* had the highest detection rate of 25/28 isolates, with only 3 isolates not possessing this gene. The other three genes, *ASA1*, *ACE*, and *gelE*, had positive detection rates of 82% (23/28 isolates), 57% (16/28 isolates), and 61% (17/28 isolates), respectively (Fig. 1).

### Virulence gene profiles

Seven distinct virulence gene profiles were detected among the 28 isolates of *E. faecium.* Isolates carrying the four virulence genes *ace-asa1-acm-gelE* accounted for the highest proportion at 46% (13/28 isolates), followed by isolates carrying the *acm* gene alone, accounting for 18% (5/28 isolates). In addition, the proportions of isolates carrying the virulence genes *asa1-acm*, *asa1-acm-gelE*, *ace-asa1-gelE*, *ace-acm-asa1*, and *asa1* alone were 14% (4/28 isolates), 4% (2/28 isolates), 7% (2/28 isolates), 4% (1/28 isolate), and 4% (1/28 isolate), respectively. Statistics relating to the virulence gene profiles of the 28 isolates of *E. faecium* are shown in Table 5.

**Table 5:** Virulence determinant profiles

Number of genes	Virulence-associated gene profile	Numbers of isolates	Prevalence %
4	*ace-asa1-acm-gelE*	13	46.43
1	*acm*	5	17.86
2	*asa1-acm*	4	14.29
3	*asa1-acm-gelE*	2	7.14
3	*ace-asa1-gelE*	2	7.14
2	*ace-asa1-acm*	1	3.57
1	*asa1*	1	3.57

*cylA* cytolysin, cpd sex pheromones, *asa1* aggregation substance, *ace/acm* adhesin to collagen, *esp* enterococcal surface protein, *gelE* gelatinase, *hyl* hyaluronidase

### Drug sensitivity profiles

**Fig. 2:**
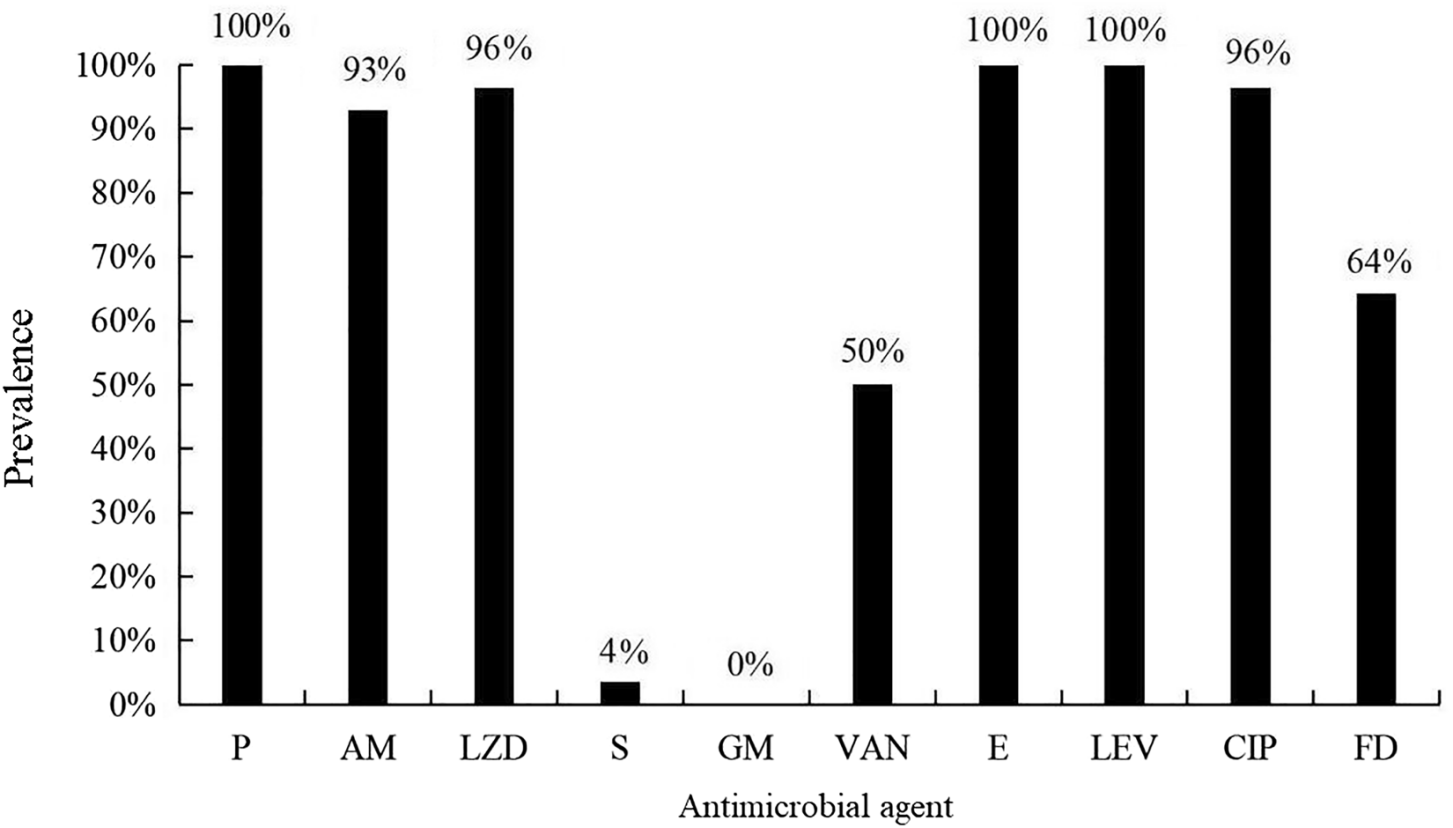
Prevalence of resistance to 10 antibiotics in 28 *Enterococcus faecium* isolates P penicillin, *AM * ampicillin, *LZD* linezolid, *S* streptomycin, *GM * gentamicin, *VAN*  vancomycin, *E* erythromycin, *LEV * levofloxacin, *CIP *ciprofloxacin, *FD* nitrofurantoin

The 28 isolates of *E. faecium* showed an antimicrobial resistance rate of > 90% to penicillin, ampicillin, linezolid, erythromycin, levofloxacin, and ciprofloxacin. Figure 2 shows the resistance of the 28 isolates of *E. faecium* to various antibiotics.

### Identification of drug resistance genes

**Fig. 3:**
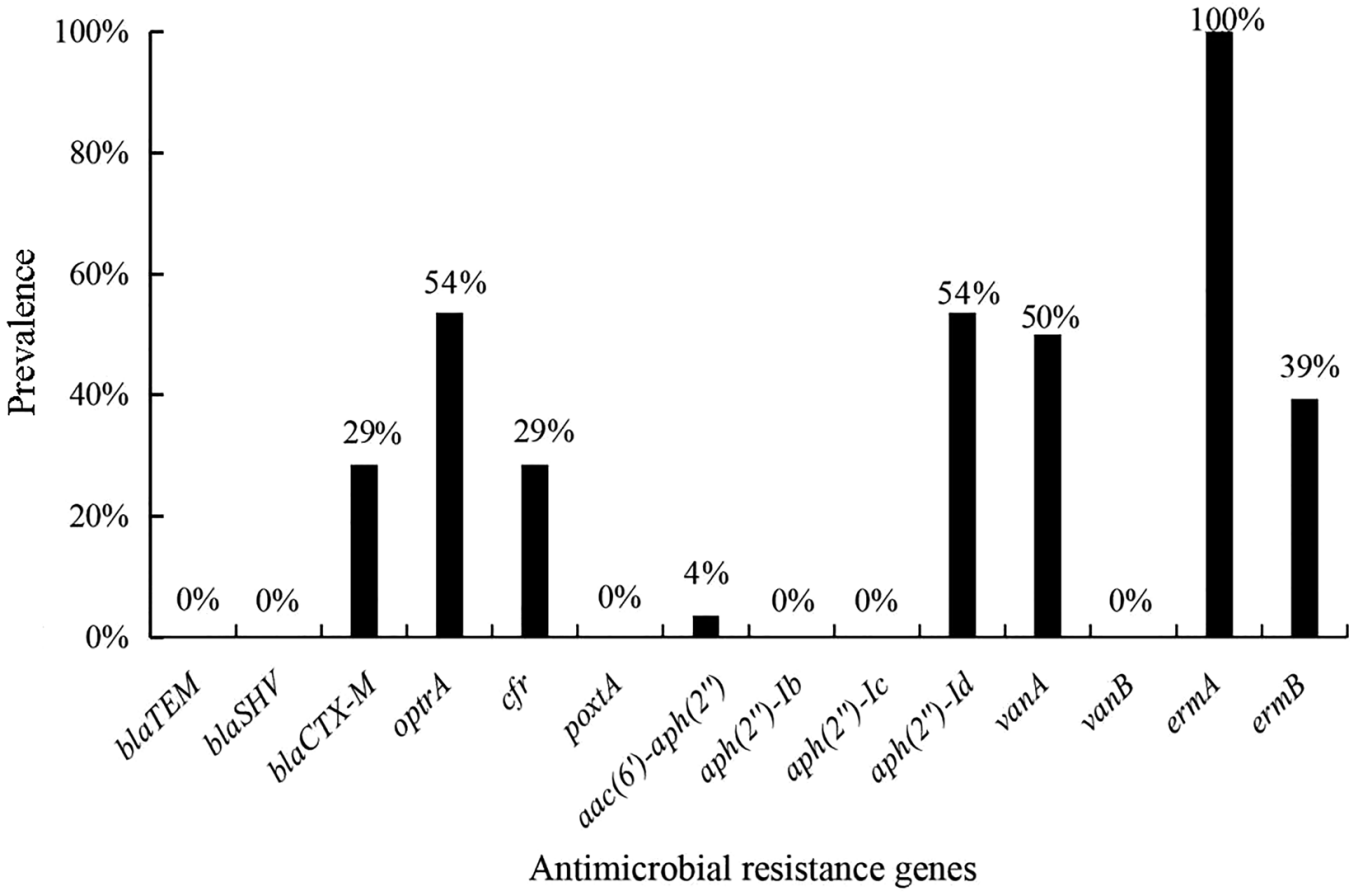
Prevalence of antimicrobial resistance genes in 28 *Enterococcus faecium* isolates Note: β-lactam resistance genes: *blaTEM*, *blaSHV* and *blaCTX-M*; Linezolid resistance gene: *cfr, optrA* and *poxtA*; Aminoglycoside resistance genes: *aac(6*′*)-aph(2*′′*)*, *aph(2*′′*)-Ib*, *aph(2*′′*)-Ic* and *aph(2*′′*)-Id*; Vancomycin resistance gene: *vanA* and *vanB*; Erythromycin resistance gene: *ermA* and *ermB*.

Eight drug resistance genes were detected in this study (Fig. 3). The β-lactam antibiotic resistance genes *bla*TEM and *bla*SHV were not detected in any of the 28 isolates of *E. faecium*. The positive detection rate for *bla*CTX-M was 29% (8/28 isolates). The positive detection rates for the linezolid resistance genes *cfr* and *optr*A were 54% (15/28 isolates) and 29% (8/28 isolates), respectively. The *poxt*A gene was not detected, and the aminoglycoside resistance gene *aac(6′)-aph(2′′)* was only detected in one isolate. The positive detection rate for *aph(2′′)-Id* was 54% (15/28 isolates). The positive detection rate for *van*A, a gene encoding resistance to the glycopeptide antibiotic vancomycin, was 50% (14/28 isolates), whereas *van*B was not detected. The positive detection rate for the *erm*A gene was as high as 100%, whereas that for *erm*B was only 39% (11/28 isolates).

## Discussion

Although studies have shown that *E. faecium* has few virulence genes, the clinical infection rate and the mortality rate for *E. faecium* infections are increasing annually. This implies that *E. faecium* may have additional pathogenic mechanisms or virulence genes that have not yet been discovered [15, 16]. We analyzed 28 isolates of *E. faecium* isolated from healthy giant pandas at the Chengdu Giant Panda Breeding Research Base in Sichuan Province to better understand the virulence genes, resistance genes, and drug resistance carried by *E. faecium*. We identified virulence genes and antibiotic susceptibility of *E. faecium* isolates from giant pandas, which provide insight into the occurrence of this pathogen and potential treatment strategies for the oral or digestive system diseases that it induces in pandas.

In this study, the virulence genes *asa1*, *ace*, *acm*, and *gelE* were detected in the *E. faecium* isolates, but *ace-asa1-acm-gelE*, *cylA*, *cpd*, *esp*, and *hyl* were not found. Our results showed that the *acm* gene was the most prevalent (25/28 isolates). Research has shown that the positive detection rate of the *cyl* gene of *E. faecium* is significantly lower than that of *E. faecalis* [13]. This is similar to our finding that no *cylA* gene was detected in any of the 28 *E. faecium* isolates. The prevalence of the *asa1* gene among *E. faecium* isolates varies greatly among studies, with some reporting no detection of the *asa1* gene [17], some reporting low *asa1* detection rates [18], and some reporting positive detection rates as high as 100% [19]. According to Baylan et al. [20], the aggregated substance gene *asa1* is linked to *E. faecium* resistance to ciprofloxacin, norfloxacin, and levofloxacin. In this study, the detection rate of *asa1* was 82%. Sex pheromones, including those encoded by the *cpd* gene, are closely related to the induction of aggregation substance, which plays vital roles in the dissemination of antimicrobial resistance and virulence genes among bacteria [21]. However, the *cpd* gene was not detected in any of the 28 isolates of *E. faecium* in this study, suggesting the possibility that other pheromone-coding genes may be present or that these isolates can be induced to express *asa1* by other substances, such as serum. Although whether the *esp* gene of *E. faecium* is linked to adhesion and biofilm formation remains to be confirmed, the detection rate for the *esp* gene is exceptionally high in clinical drug-resistant isolates.

Resistance to ampicillin, ciprofloxacin, pentaebacterium, and glycopeptide antibiotics is highly correlated with the presence of the *esp* gene in *E. faecium* [22, 23]. Erdem et al. [24] reported an *esp* gene detection rate of up to 90% among drug-resistant *E. faecium* isolates. The *hyl* gene may also alter *E. faecium* resistance to vancomycin. In one study of *E. faecium*, 157 (81%) of the 193 *esp-*positive isolates and 85 (83%) of the 102 *hyl-*positive isolates were vancomycin-resistant, and the *hyl* gene was frequently found alongside the *esp* gene [25]. In our current study, the *esp* and *hyl* genes were not detected in any of the 28 *E. faecium* isolates tested. The *ace* gene was previously assumed to be exclusive to *E. faecalis*; however, new research has confirmed that the *ace* gene is harbored by a small number of other isolates [22]. By contrast, the *acm* gene has a high positive detection rate among *E. faecium* isolates. Nallapareddy et al. [26] investigated 90 isolates of *E. faecium* from various sources and found *acm* positivity rates of up to 99% (89 isolates). The positive rate of detection of the *ace* gene was 57.14% (16/28 isolates) among our isolates, which may reflect horizontal gene transfer between *E. faecium* and *E. faecalis*. The positive detection rate for the *acm* gene was 89%; the gene was not detected in three isolates. The prevalence of the *gelE* gene, which encodes gelatinase, has been shown to be variable, ranging from 19.6% to 80.4% of isolates; however, its link to *E. faecium* resistance is unknown. In this study, the *gelE* gene had a positive detection rate of 61%.

In this study, most of the *E. faecium* isolates were shown to be multidrug-resistant yet sensitive to high doses of aminoglycosides. Eight resistance genes were identified, with the number of resistance genes in individual *E. faecium* isolates ranging from one to six. Multidrug resistance is defined as resistance to at least one medication in each of three or more antimicrobial classes. Multidrug resistance has been found to be prevalent in *E. faecium*, with isolates showing resistance to four or more medications. Previously, the effectiveness of antibiotic treatment of *E. faecium* infections was improved by combining antibiotics [27]. However, as the abundance of *E. faecium* has increased, the use of antibiotics such as penicillin and high concentrations of aminoglycosides, quinolones, glycopeptides, and even linezolid has become less effective [28]. As a result, finding new antimicrobials with high antibacterial properties and low drug resistance is critical.

The natural resistance of *E. faecium* to β-lactams is considered low to moderate; strong resistance is dependent on the production of β-lactamase. The 28 *E. faecium* isolates tested in the present study were highly resistant to β-lactam antibiotics, with resistance rates of 100% for penicillin and 93% for ampicillin, and the *blaCTX-M* gene expressing CTX-M had a positive detection rate of 29%. The occurrence of clinical high-level gentamicin resistance indicates that gentamicin and cell wall–active antibiotics, such as ampicillin and vancomycin, have lost their synergy. In China, high-level gentamicin resistance (64.7% (33/51 isolates)) was reported in hospital enterococci [29]. In the present study, 28 isolates of *E. faecium* showed high sensitivity to high concentrations of gentamicin and streptomycin, with resistance rates of 4% and 0%, respectively. The aminoglycoside resistance genes detected included *aac(6′)-aph(2′′)* and *aph(2′)-Id*, with the *aac(6′)-aph(2′′)* gene being detected in only one isolate. This shows that high dosages of aminoglycosides may still be effective against enterococcal infections in captive pandas. Interestingly, the novel antimicrobial drug linezolid was virtually ineffective against the 28 isolates in this study, but the detection rates of the resistance genes *cfr* and *optrA* were 54% and 29%, respectively. These rates were not as high as those for their resistant phenotypes, and the *poxtA* gene was not detected. This finding indicates that the *E. faecium* isolates studied here may harbor different resistance mechanisms. Vancomycin is a first-line medicine for the treatment of enterococcal infections, and the emergence of vancomycin-resistant enterococci is posing a significant clinical issue [30]. Iseppi et al. [31] studied the resistance of *Enterococcus* sp. and *Enterobacter* sp. isolated from 100 stool samples of 50 people, 25 dogs, and 25 cats. The authors found that *E. faecium* was the most common microorganism in the examined humans, dogs, and cats and that all vancomycin-resistant isolates were *vanA*-positive [31]. Vancomycin was found to be effective against half of the *E. faecium* isolates in this study, with a 50% positive detection rate for the *vanA* gene, suggesting that vancomycin could be used in combination with other medications to improve outcomes.

Mutations in the *parC* and *gyrA* genes are the main cause of *E. faecium* resistance to quinolone antibiotics. Brisse et al. [32] partially sequenced the *parC* and *gyrA* genes of 73 ciprofloxacin-resistant and 6 ciprofloxacin-sensitive *E. faecium* isolates; they found that topoisomerase IV was the main target of ciprofloxacin in *E. faecium* and that the effect of the *gyrA* mutation on the resistance of *E. faecium* to ciprofloxacin was limited. The principal targets of sparfloxacin and norfloxacin are DNA DNA gyrase and topoisomerase IV, respectively [33].

In this study, the *E. faecium* isolates were highly resistant to quinolones, with a 100% levofloxacin resistance rate and a 96% ciprofloxacin resistance rate. The early drug nitrofurantoin has attracted renewed interest as a result of the emergence of vancomycin-resistantenterococci; however, the efficacy of nitrofurantoin was poor in this study, and isolates showed a resistance rate of 64%; this should be taken into account in the clinical setting.

## Conclusions

This is the first study to focus on the virulence and resistance of oral-derived *E. faecium* in giant pandas. Our study sheds light on the prevalence of multidrug resistance and the virulence genes in *E. faecium*; it also highlights the need to monitor antibiotic resistance in more *E. faecium* isolates from captive giant pandas. We found that the isolates were sensitive to high concentrations of aminoglycosides, vancomycin, and nitrofurantoin, findings that may provide a reference for the rational management of *Enterococcus* infections in giant pandas. Although these results will help to establish strategies for prevention and surveillance of antimicrobial resistance in captive giant pandas, further exploration may be needed to elucidate the rational use of antibiotics for treatment of captive giant pandas and other protected animals.

## Data Availability

The datasets used and/or analysed during the current study are available from the corresponding author on reasonable request. The gene sequences were deposited in GenBank under Accession No. OQ255818-OQ255845.
